# A Dynamic Relationship between Environmental Degradation, Healthcare Expenditure and Economic Growth in Wavelet Analysis: Empirical Evidence from Taiwan

**DOI:** 10.3390/ijerph17041386

**Published:** 2020-02-21

**Authors:** Cheng-Feng Wu, Fangjhy Li, Hsin-Pei Hsueh, Chien-Ming Wang, Meng-Chen Lin, Tsangyao Chang

**Affiliations:** 1 School of Business Administration, Hubei University of Economics, Wuhan 430205, China; wuchengfeng@hbue.edu.cn (C.-F.W.); mengchen0608@gmail.com (M.-C.L.); 2 Research Center of Hubei Logistics Development, Hubei University of Economics, Wuhan 430205, China; 3 Department of Finance, School of Finance, Hubei University of Economics, Wuhan 430205, China; fangjhy@hbue.edu.cn; 4 School of Finance, Hubei University of Economics, Wuhan 430205, China; xuexinbei@hbue.edu.cn; 5 School of Economics and Trade, Hubei University of Economics, Wuhan 430205, China; 6 Department of Finance, School of Finance, Feng Chia University, Taichung 40724, Taiwan; tychang@mail.fcu.edu.tw; 7 CTBC Business School, Tainan 709, Taiwan

**Keywords:** wavelet analysis, healthcare expenditure, environmental degradation, economic growth, Taiwan

## Abstract

This paper analyzes the co-movement and causal linkages between environmental pollution and healthcare expenditure, taking economic growth as a control variable by using wavelet analysis for Taiwan over the period 1995 Q1–2016 Q4. The results show that there exists co-movement and causality between environmental pollution and healthcare expenditure at different frequencies and times. The changes in the relationships of the two variables are observed in certain events such as the period of the expansion stage, the policy of environmental pollution, and the issue of the National Health Insurance Integrated Circuit card (NHI-IC) in Taiwan. In the short-term, positive causality runs from healthcare expenditure to environmental pollution before 2004, while negative causality runs from healthcare expenditure to environmental pollution before 2007 in the long-term. After adding economic growth as a control variable, positive causality runs from healthcare expenditure to environmental pollution in the period 2009–2011 in the short-term, while negative causality running from healthcare expenditure to environmental pollution is shown in 2008 in the long-term. The results indicate that “higher government health expenditure leading to higher demand for environment quality” exists in different sub-periods and the argument may concern the factor of economics in the long-term. The positive healthcare lead in the short-term may be based on economics in the expansion stage. Also, the issue of NHI-IC possibly affects the dynamic relationship between healthcare expenditure and environmental pollution without considering economics. Based on empirical analysis, certain policy and managerial implications are addressed for decision-makers at macroeconomic and microeconomic levels.

## 1. Introduction

In recent decades, the relationships between economic growth, environmental deterioration and health spending have received greater focus in literature in the context of both developed and developing nations. These relationships among economic growth, environmental deterioration and health spending are varied, complicated and significant.

Increased economic expansion, urbanisation and industrialisation within both developed and developing nations have caused the significant worsening of air quality as a result of the emission of a variety of different air pollutants into the environment [1]. Human actions, such as the burning of fossil fuels utilised for generating electricity as well as the transportation sector, are the primary contributors to environmental pollution [2]. Dangerous air pollutants or chemicals emitted into the environment by various natural or anthropogenic processes can lead to detrimental impacts on people’s health [3,4,5]. Hence, authorities may allocate resources targeted at the improvement of the prevention of environmental pollution [6]. While governments must take into account the balance between the expenditure allocated to enhancing health and the accumulation of human capital and technology, it is evident that improvements made to human health can facilitate the growth of the economy as a result of the enhanced well-being of the population [7].

The pairwise relationship that exists among the three issues presented in the study has been examined in the literature. The association between economic growth and health, in particular, has been the focus of numerous studies [8,9,10]. The majority of previous researchers have concentrated on how to estimate the size of the income elasticity of healthcare, in addition to the policy implications in terms of the funding and distribution of healthcare resources. Recently conducted studies have also presented a link between health and environmental deterioration in order to examine empirical evidence [11,12,13]. The majority of previous studies have concentrated on the impact of CO_2_ emissions on health spending. A limited number of research have specifically concentrated on the bi-directional causality between environmental degradation and health costs. Lastly, based on the pioneering work of Grossman and Kruger [14], multiple researchers have investigated the Environmental Kuznets Curve between economic growth and environmental degradation [15,16,17,18,19].

Air quality has developed into a significant national problem in Taiwan since the 1990s. It was determined that mobile emissions represent one of the primary contributors to air pollution in this area in comparison to industrial plants. For instance, estimations for 2017 indicated that mobile emissions contributed a total of 74% of the overall carbon monoxide (CO) emissions [20]. As a result of Taiwan’s rapidly developing economy and the subsequent demands for improvements to be made to people’s standard of living, the volume of motorised vehicles has increased exponentially in the last 20 years. Between December 1995 and the end of May 2019, the number of small passenger vehicles and motorbikes grew from 4.7 to 8.1 million, which represents a 1.7-fold increase, whereas the number of motorcycles rose from 8.5 to 13.9 million, representing a 1.6-fold increase [21]. According to statistics taken from the end of May 2019, the number of motorbikes per person and the number of small passenger vehicles per person were 0.93 and 0.34, respectively. As a result of the exponential growth in the number of motorised vehicles in the country, the problems of environmental pollution and sanitation resulting from inadequate processes used in their disposal have attracted increased attention.

After the government introduced the scheme of National Health Insurance in 1995, users, no matter the rich or poor, were able to enjoy the resources in the scheme in which users can undergo health examinations and cures with partial subsidies from the scheme, such that National Health Insurance benefited everyone in the society. The government often subsidises those who are not rich in society; for example, in Taiwan, farmers are endorsed 70% and low-income families are endorsed 100% for the payment [22]. As a result, those users who cannot relatively afford huge medical fees benefit from the scheme. After introducing NHI, the people in the middle or bottom of the economic ladder, who now possessed better physical and mental situations, contributed to the economy, such that the positive externalities in the awareness of quality of life were shown. The government in Taiwan was devoted to decrease inequality for residents and establish a better environment for life based on the launch of NHI and the implementation of environmental protection policy.

There is sufficient empirical evidence demonstrating the pairwise association between the three primary issues, namely, environmental pollution and healthcare spending, environmental pollution and economic growth, and healthcare spending and economic expansion. Limited studies have focused on the casualty between environmental pollution and healthcare spending; also, they have investigated the issue utilizing panel data based on a regional context through employing the time series technique [23,24]. Particularly, healthcare spending has direct or indirect impact on environmental pollution depending on the policy of government [23]. Apergis et al. [25] showed the causal effect runs from environmental degradation to healthcare expenditure in U.S. states. Taiwan is one of the few countries that implement an NHI subsided by government spending; also, Taiwan is taken as an emerging country and the development of economics and policy on healthcare and environment may be different from most of other developing countries.

To the best of the authors’ knowledge, the present study represents the first attempt to examine the co-movements between healthcare spending and environmental pollution, where economic growth has been used as the control variable for Taiwan through the application of the time and frequency domain approach. The contribution that this research makes to the literature can be viewed from two perspectives. Firstly, we utilise the wavelet approach, which has the capacity to investigate the dynamics between healthcare spending and environmental pollution, where economic growth is taken as the control variable in both long-term and short-term. The benefit of wavelet analysis is that it takes into account both time and frequency jointly in one structure in the examination of the co-movement in time series of healthcare spending and environmental pollution in a time-frequency band space. From the economic perspective, time-frequency domain techniques allow cyclical data to be extracted from a time series, which can be highly beneficial in the evaluation of the impacts of distinct socio-economic situations in terms of the different positions for environmental and healthcare policies, due to the fact that it provides recommendations in regard to the drive and timing of policy actions that traditional time-series methods are unable to capture completely. Hence, analysis based on the results is comparatively comprehensive in regard to the relationships between the given variables. Secondly, the present study could be particularly beneficial for Taiwanese policymakers responsible for healthcare and environmental issues and will offer a certain degree of imperative understanding to other economies with a level of development and healthcare expenditure similar to Taiwan in both the short and long term.

The study is separated into five sections. Section 2 presents a review of the literature. The data and methodology used are described in Section 3. Section 4 presents the empirical findings and Section 5 concludes the research.

## 2. Literature Review

The aim of this study is to empirically investigate the co-movements among healthcare spending and environmental pollution for Taiwan, where economic growth is taken as the control variable. The pairwise association among the primary study variables will be discussed on the basis of the following strands.

### 2.1. Environment and Economic Growth

First of all, the Environmental Kuznets Curve has been proposed by Grossman and Krueger [14] who addressed the relationships between the environment and economics as an inverted hump-shaped curve. They indicated that environmental deterioration increases proportionally to income, and then reaches a plateau at the point that income reaches a particular threshold, at which point it starts to decrease.

Hallicioglu [26] examined the nature of the causality between economic expansion and carbon dioxide emissions in a sample of Turkey by utilising the ARDL bounds approach from 1960 to 2005. They indicated that the time series are affected mutually. Wang [27] investigated the causal effect between carbon dioxide emissions and economic growth for 138 nations for the timespan 1971–2007 by using the error correction model, and the findings supported the feedback hypothesis. Jebli et al. [17] determined that two-way causalities existed in the long-run between per capita CO_2_ emissions and GDP in 25 OECD countries through using panel data for the period between 1980 and 2010. Utilising a sample comprising 16 Chinese provinces located in three primary regions for the period 1985–2012, Peng et al. [19] determined that GDP was Granger-causing carbon dioxide emissions in 15 of the provinces studied, while a bidirectional causality was found to exist between GDP and carbon dioxide emissions for Shanxi province. Pata [18] explored the dynamic associations between economic growth and environmental deterioration in the context of Turkey for the period between 1974 and 2014, and the findings indicated that economic growth leads to significant environmental deterioration. Cai et al. [16] examined the relationship among carbon dioxide emissions, economic growth and renewable energy consumption via the application of the newly introduced bootstrap ARDL bounds test with structural breaks. Their findings confirmed there exists the association between CO_2_ emissions and economic growth in the long run for the G7 nations. In summary, past research has presented opposing evidence in terms of the EKC hypothesis for both individual country and cross-country studies.

### 2.2. Economic Growth and Health Expenditures

Secondly, this section focuses on the relationship between economic growth and health expenditures. Most of the research has focused on measuring the magnitude of the income elasticity of healthcare, in addition to the policy ramification for the funding and the efficiency in the allocation of healthcare resources.

Ayuba [28] utilised various estimation methods to examine the causality between healthcare spending and economic growth. This study focuses on Nigeria. The outcomes revealed that a unidirectional causality exists flowing from economic growth to healthcare expenditures. Chen [9] applied the wavelet technique to determine the lead-lag linkage among economic growth and health development in the United States. Liu et al. [10] explored the nexus between health progress and business cycles, taking the United States as a sample by using the mixed-frequency vector auto-regressive model. Albulescu et al. [8] examined the level of convergence in healthcare spending for six EU member countries for the time period covering 1972 to 2013. The findings indicated that no significant convergence occurred with regard to the ratio of healthcare spending to GDP.

### 2.3. Environment and Health Expenditures

The third research strand on this issue offers empirical evidence regarding the nexus between the environment and healthcare spending. However, there has been minimal focus within the academic community in terms of this research strand in comparison to the two strands mentioned above. The majority of previous researchers have concentrated on the causal effect of CO_2_ emissions on healthcare spending. Not so many empirical investigations have investigated the bi-directional causality between the environment and healthcare spending.

In their research focused on 30 Chinese provinces, Lu et al. [29] determined that CO_2_ and other pollutants that impact the environment had a negative effect on human health. In the study conducted by Yazdi et al. [30], a positive casualty between CO_2_ and other pollutants to healthcare spending for Iran by utilising cointegration as well as the ARDL approach was investigated. Apergis et al. [25] studied CO_2_ emissions at the individual state level for 50 states and the resulting effects on healthcare spending. They determined that CO_2_ had a positive effect on healthcare expenditure and further analysis revealed that this impact was more powerful for states whose healthcare expenditures were higher. Khoshnevis Yazdi and Khanalizadeh [11] examined the contribution of environmental quality and economic growth in determining healthcare expenditures for countries located in the Middle East and North Africa for the timespan 1995–2014 by utilising the ARDL model. The findings revealed that increased economic growth elevated healthcare spending, meaning that economic growth is a significant indicator of health spending. Matthew et al. [12] utilised the ARDL approach for the timespan covering 1985 to 2016 to investigate the impact of greenhouse gas (GHG) emissions on life expectancy for Nigeria. They indicated that GHG emissions have a negative relationship with life expectancy, but a positive relationship with mortality rates. According to the results presented in the study by Moosa and Pham [14], the relationship between healthcare spending and environmental deterioration could be negative or positive, based on the per capita income levels. In summary, few studies have been conducted on the nexus between CO_2_ emissions and healthcare spending due to the fact that researchers have given this subject less attention. The positive and causal association between healthcare spending and CO_2_ emissions has been validated by past panel-based research by [23,24,31].

A minimal number of studies have focused on the relationship between air quality, healthcare spending and economic growth. Chaabouni and Abdnnadher [32] investigated the causality between environmental quality, economic growth, and health spending in the context of Tunisia. They applied Granger causality tests and suggested the presence of a robust two-way causality between the aforementioned variables for the period between 1960 and 2008. Chaabouni et al. [23] demonstrated that a two-way causality exists between economic growth and carbon dioxide emissions, and between healthcare spending and economic growth based on a global panel approach, as well as a unidirectional causality running from carbon dioxide emissions to health spending, apart from in countries with low income. Mehrar et al. [33] examined the nexus among economic growth, health expenditure, and environmental quality by applying a panel cointegration approach. The data are from 114 developing nations for the timespan of 1995 to 2007. The empirical findings supported unidirectional causality flowing from carbon dioxide emissions to economic growth, as well as from economic growth to healthcare spending.

In summary, the majority of prior research has concentrated on the pairwise association between the three primary issues, namely “environment and economic growth”, “economic growth and healthcare spending” and “environmental quality and healthcare spending”. Additionally, multiple studies have explored the nexus among the three primary issues using the panel cointegration technique based on a regional context. Furthermore, for individual economies, the majority of past studies have investigated the relationship among these variables via the application of time domain methods. Nevertheless, it is evident that in order to examine the three primary issues and to make structured policy recommendations for Taiwan, it is necessary to explore the dynamic association between health spending and environmental pollution, where economic growth is taken as the control variable through wavelet analysis. Wavelet analysis is a time and frequency domain technique. Instead of time domain methods, policy makers employ the time and frequency domain method to comprehensively focus on both long- and short-term analysis.

## 3. Wavelet Analysis

During the mid-1980s, wavelet analysis was developed as another option to the familiar Fourier analysis. Time-localised information is totally ignored in the Fourier transform despite the fact that such analysis may unmask the relationships over various frequencies through spectral methods. Furthermore, the appropriateness of the Fourier analysis is limited to the stationary time series. Contrastingly, we are able, through wavelet analysis, to assess the spectral attributes of a time series (being a time function) and subsequently to remove localised information in frequency areas as well as in time domains [34]. Moreover, in the case of the time series in question being either locally stationary or non-stationary, wavelet analysis is appreciably better than the Fourier analysis [35].

### 3.1. The Continuous Wavelet Transform

In wavelet analysis, initially, the wavelet transform fragments a time series into extended and transformed types of a specified and well-localised “mother wavelet” within frequency and time areas. This enables the expansion of the series into a time-frequency space where highly-instinctive observations are made of the frequency-varying and time-varying oscillations. There are frequently two wavelet transforms classifications, namely, discrete wavelet transforms (DWT) and continuous wavelet transforms (CWT). The DWT aids data compression as well as noise reduction, whereas the CWT is of greater assistance for data self-similarity recognition and feature extraction [36,37]. The CWT is effectively applied broadly in the financial and economic fields [34,38,39].

We can define the CWT of a time series $x\left(t\right)\in L^{2}\left(\mathbb{R}\right)$ concerning the mother wavelet Ψ(t) as the inner product of x(t) within the wavelet daughter’s family $\Psi_{\tau ,s}\left(t\right)$:

(1) $$W_{x;\Psi }\left(\tau ,s\right)=\langle x\left(t\right),\Psi_{\tau ,s}\left(t\right)\rangle =\int_{-\infty }^{+\infty }x\left(t\right)\Psi_{\tau ,s}{}^{*}\left(t\right)dt$$

in which the asterisk (*) indicates complex conjugation, meaning that $\Psi_{\tau ,s}{}^{*}\left(t\right)$ represents the daughter wavelet’s complex conjugate functions, $\Psi_{\tau ,s}\left(t\right)$. As aforementioned, the mother wavelet Ψ(t) is the source of $\Psi_{\tau ,s}\left(t\right)$ in the dissolution process where $\Psi_{\tau ,s}\left(t\right)=\frac{1}{\sqrt{\left|s\right|}}\Psi \left(\frac{\left(t-\tau \right)}{s}\right)$, τ,s∈ℝ, s≠0. When the wavelet scale parameter differs, s suggests extending (if |s|≻1) or compressing (if |s|≺1) the mother wavelet Ψ(t) over frequencies, in the course of translating along the localised time index τ. This suggests moving the wavelet’s location Ψ(t) within the correct time. Through this action, it is possible to build a picture which demonstrates the amplitude of any aspects in x(t) as against the scale and the means by which this amplitude develops in the course of time [40]. Furthermore, this is referred to as continuous wavelet transform because both s and τ are real values which are subject to perpetual variation with the constraint s≠0, and $W_{x;\Psi }\left(\tau ,s\right)$.

It is necessary for Ψ(t) to satisfy two essential conditions in order to be a CWT mother wavelet, namely $\Psi \left(t\right)\in L^{2}\left(\mathbb{R}\right)$ and the requirement referred to as the “admissibility condition”, which may be expressed as

(2) $$0≺C_{\Psi }=\int_{-\infty }^{+\infty }\frac{{\left|\hat{\Psi }\left(f\right)\right|}^{2}}{\left|f\right|}df≺+\infty$$

in which $\hat{\Psi }\left(f\right)$ represents the mother wavelet’s Fourier transform, Ψ(t) and f indicates the Fourier frequency [41]. It is obvious when examining this formula that $C_{\Psi }$ is independent of f and exclusively established by the wavelet Ψ(t), thereby implying $C_{\Psi }$ to be a constant for each given mother wavelet function; consequently, it is referred to as the “admissibility constant”. The admissibility condition (2) is significant in that it ensures the likelihood of recovering time series x(t) from its CWT, $W_{x;\Psi }\left(\tau ,s\right)$ as shown below:

(3) $$x\left(t\right)=\frac{1}{C_{\varphi }}\int_{-\infty }^{+\infty }\left[\int_{-\infty }^{+\infty }W_{x;\Psi }\left(\tau ,s\right)\Psi_{s;\tau }\left(t\right)d\tau \right]\frac{ds}{s^{2}},\;s\neq 0$$

By using this, we may proceed from x(t) to the CWT and then return to x(t). Therefore, it is justifiable to accept that x(t) and $W_{x;\Psi }\left(\tau ,s\right)$ are basically two different “representations of the same mathematical entity” (see Aguiar-Conraria et al. [34], p. 2868.). It is more significant that the initial energy of x(t) may be upheld by its wavelet transform, as shown below.

(4) $${\left\|x\right\|}^{2}=\int_{-\infty }^{+\infty }{\left|x\left(t\right)\right|}^{2}dt=\frac{1}{C_{\Psi }}\int_{-\infty }^{+\infty }\left[\int_{-\infty }^{+\infty }{\left|W_{x;\Psi }\left(\tau ,s\right)\right|}^{2}d\tau \right]\frac{ds}{s^{2}}$$

in which ${\left\|x\right\|}^{2}$ represents the energy of x(t).

There are many different kinds of mother wavelets for various purposes. These include Daubechies, Haar, Mexican hat and Morlet. The Morlet wavelet, which was introduced by Goupillaud et al. [42], is the most frequently applied mother wavelet for the purpose of feature removal. It may be represented in a simple form as

(5) $$\Psi \left(t\right)=\pi^{-\frac{1}{4}}e^{i\omega_{0}t}e^{-\frac{t^{2}}{2}}$$

in which $\pi^{-\frac{1}{4}}$ guarantees the mother wavelet’s unity energy. Furthermore, $\omega_{0}$ indicates the dimensionless frequency, and in practice, it is normally valued at six. The reason for this is that this value can guarantee the Morlet wavelet to be close to an analytic wavelet and that it facilitates the interpretation of the connection between the scale s and the Fourier frequency f.

### 3.2. The Wavelet Power Spectrum

The wavelet power spectrum of a time series x(t), according to wavelet theory, is basically shown as ${\left|W_{x;\Psi }\left(\tau ,s\right)\right|}^{2}$, being named as the auto-wavelet power spectrum. We can explain this as an assessment of the local variance at each frequency for x(t). Hudgins et al. [43] implemented the cross-wavelet transform of two time series, x(t) and y(t), which are defined as $W_{xy;\Psi }\left(\tau ,s\right)=W_{x;\Psi }\left(\tau ,s\right)W^{*}{}_{y;\Psi }\left(\tau ,s\right)$ in which their cross-wavelet power spectrum is correspondingly expressed as ${\left|W_{xy;\Psi }\left(\tau ,s\right)\right|}^{2}={\left|W_{x;\Psi }\left(\tau ,s\right)\right|}^{2}{\left|W^{*}{}_{y;\Psi }\left(\tau ,s\right)\right|}^{2}$, thereby offering an assessment of the local covariance between x(t) and y(t) at each frequency. Wavelet power is indicated by the colours in the wavelet power spectrum plots, in which red represents high power and blue represents low power. As aforementioned, an assessment of the local volatility is offered by wavelet power; likewise, the colours represent the local volatilities.

### 3.3. The Wavelet Coherency and Phase Difference

To conduct an analysis of the dynamic association between health expenditure and environmental degradation in Taiwan, greater attention ought to be applied to the phase difference and wavelet coherency. The process begins with wavelet coherency, and in order to calculate this, we apply the cross-wavelet spectrum and the auto-wavelet spectra as shown below:

(6) $$R_{xy}^{2}\left(\tau ,s\right)=\frac{{\left|S\left(s^{-1}W_{xy;\Psi }\left(\tau ,s\right)\right)\right|}^{2}{\left|W^{*}{}_{y;\Psi }\left(\tau ,s\right)\right|}^{2}}{S\left(s^{-1}{\left|W_{x;\Psi }\left(\tau ,s\right)\right|}^{2}\right)S\left(s^{-1}{\left|W_{y;\Psi }\left(\tau ,s\right)\right|}^{2}\right)}$$

We observe, at this point, that the wavelet coherency being studied is shown as a squared type having similarities with past studies [34,36,39]. Following application by a smoothing operator *S*, the squared wavelet coherency produces a quantity between zero and one within a time-frequency space. In wavelet coherency plots, this is indicated by colours, in which red represents a powerful association, whereas blue represents a weak association. Consequently, wavelet coherency enables a three-dimensional analysis which may regard frequency and time components simultaneously in addition to the strength of correlation [37]. This consequently assists in the recognition of the local connection between health expenditure and environmental degradation in Taiwan as well as the recognition of structural transformations in the course of time, in addition to the long-run and short-run relationships over frequencies.

It is impossible to differentiate between positive and negative links since the wavelet coherency is squared. It is therefore necessary to phase the difference tool to existing positive or negative suggestions for associations and lead-lag links between series. The complexity of the Morlet wavelet function leads to the complexity of the CWT regarding this kind of mother wavelet, which may be separated into a real portion and an imaginary portion. Consequently, according to Bloomfield et al. [44], we can define the phase difference between x(t) and y(t) as

(7) $$\phi_{xy}=\tan^{-1}\left(\frac{ℑ\left\{S\left(s^{-1}W_{xy;\Psi }\left(\tau ,s\right)\right)\right\}}{ℜ\left\{S\left(s^{-1}W_{xy;\Psi }\left(\tau ,s\right)\right)\right\}}\right),\;\mathrm{with}\;\varphi_{xy}\in \left[-\pi ,\pi \right]$$

in which ℑ and ℜ indicate the imaginary and real portions of the smoothed cross-wavelet transform, respectively. Moreover, in accordance with Voiculescu and Usoskin [45] and Aguiar-Conraria and Soares [46], it is a simple task to convert the phase difference into the immediate time lag between x(t) and y(t) in that

(8) $${\left(\Delta t\right)}_{xy}=\frac{\phi_{xy}}{2\pi f}$$

in which 2πf is the angular frequency regarding the time scale s, in the sense that the Fourier frequency f is normally given by $f=\frac{\omega_{\Psi }}{2\pi s}$. It is noteworthy that the frequency $\omega_{\Psi }$ indicates the mother wavelet’s frequency, namely the Morlet wavelet’s dimensionless frequency $\omega_{0}$. By applying $f=\frac{\omega_{\Psi }}{2\pi s}$ with the specific selection of $\omega_{0}=6$, we obtain $f=\frac{6}{2\pi s}\approx \frac{1}{s}$. Consequently, the time lag ${\left(\Delta t\right)}_{xy}$ is ultimately given by

(9) $${\left(\Delta t\right)}_{xy}=\frac{\varphi_{xy}\cdot s}{2\pi }$$

The phase variations in this paper are shown as arrows in the wavelet coherency plots. Arrows which point to the right-hand direction indicate that x(t) and y(t) are in phase (or positively associated), whereas arrows which point to the left-hand direction indicate that x(t) and y(t) are out of phase (or negatively associated). Furthermore, arrows which point towards other directions indicate leads or lags between them. For instance, arrows which point in an upwards direction indicate that x(t) leads y(t) by one-quarter of the corresponding measurement and that it lags behind y(t) by three-quarters of the corresponding measurement. It is also worthy of mention that phase differences may imply causality between x(t) and y(t) [36,47].

### 3.4. Data

**Figure 1:**
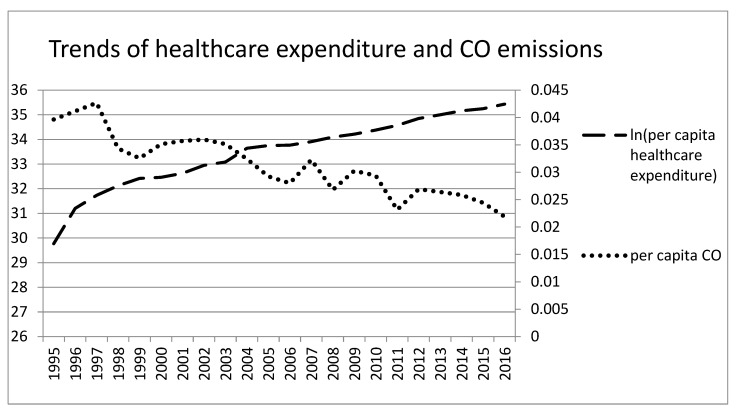
Trends of healthcare expenditure and CO emissions in Taiwan.

**Figure 2:**
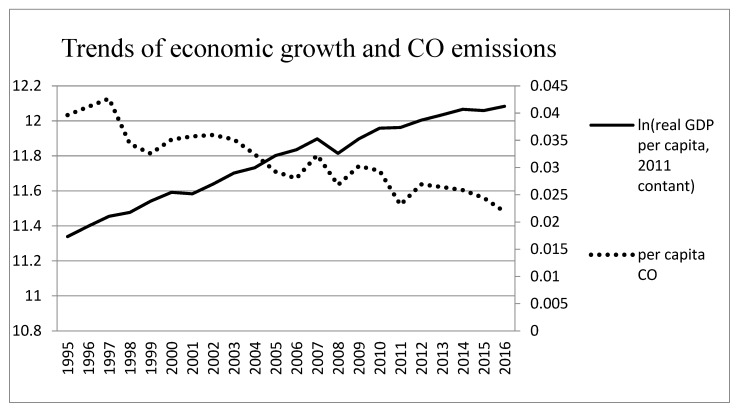
Trends of economic growth and CO emissions in Taiwan.

**Figure 3:**
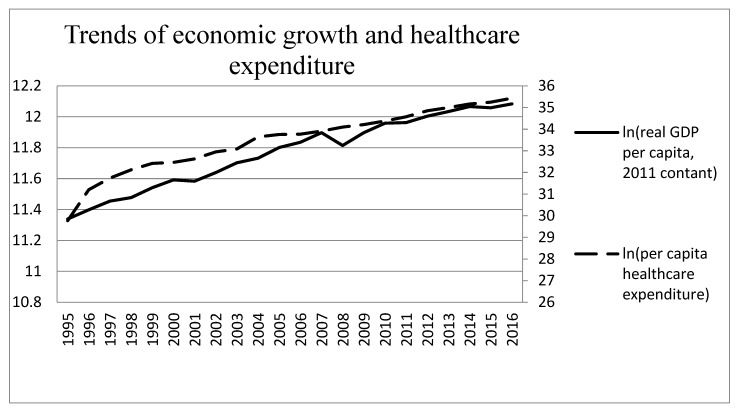
Trends of economic growth and healthcare expenditure in Taiwan.

Our empirical study uses the quarterly time series data on per capita carbon monoxide (CO) emissions (in parts per million, ppm), real GDP per capita, and healthcare expenditure per capita for the 22-year period from 1995 Q1 to 2016 Q4 in Taiwan. The data of CO is from the Environmental Protection Administration in Taiwan. The data of real GDP per capita is from national statistics in Taiwan. Real GDP per capita is measured in New Taiwan Dollars (NTD), considering 2011 prices. The data of healthcare expenditure is from the Ministry of Health and Welfare in Taiwan. We transformed the data including real GDP per capita and healthcare expenditure per capita into natural logarithms. The historical trends of per capita CO emissions, real GDP per capita, and healthcare expenditure per capita for Taiwan are illustrated in Figure 1, Figure 2 and Figure 3; dashed line, dotted line and solid line represent healthcare expenditure per capita, per capita CO emissions and real GDP per capita, respectively.

Figure 1, Figure 2 and Figure 3 show the time-series plot of healthcare expenditure and CO emissions, economic growth and CO emissions, as well as economic growth and healthcare expenditure in Taiwan. The results show that a significant movement pattern is in the association between the aforementioned variables in each figure. Regarding the association between healthcare expenditure and CO emissions as well as economic growth and CO emissions, the results show that the aforementioned variables are negatively correlated in Taiwan during certain sub-periods while the relationship between economic growth and healthcare expenditure is positively correlated. Although the time trend is shown in the figures, it is not clear enough to identify correlations or lead-lag relationships between healthcare expenditure and CO emissions. Thus, the wavelet analysis tools are applied to investigate the time- and frequency-varying relationships among the aforementioned variables for Taiwan.

## 4. Empirical Results and Discussions

**Figure 4:**
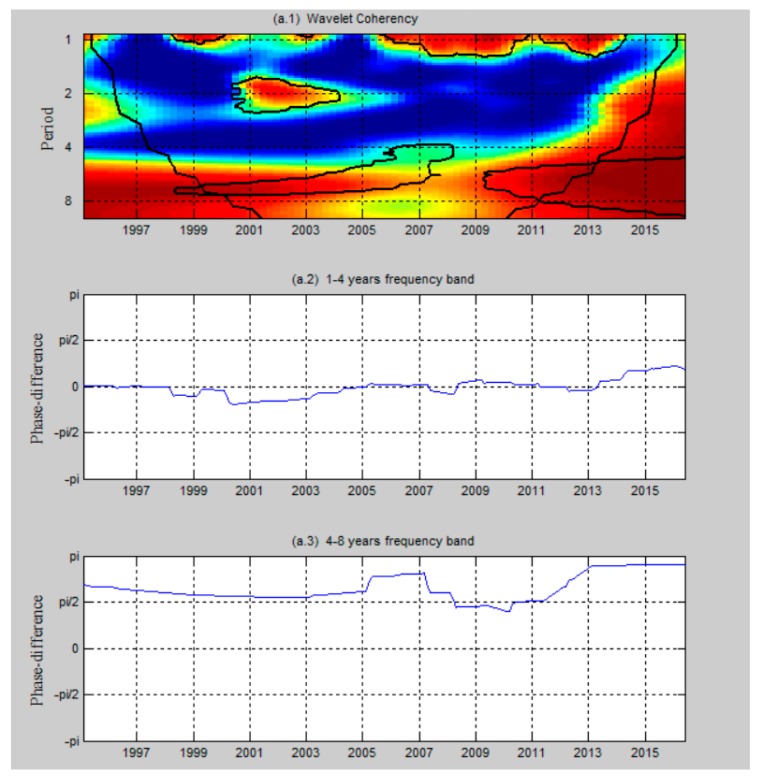
The wavelet coherency (**a.1**) and phase difference (**a.2** and **a.3**) between per capita CO emissions and healthcare expenditure per capita. The *y*-axis refers to the frequencies (measured in years); the *x*-axis refers to the time period over the period 1995–2016.

**Figure 5:**
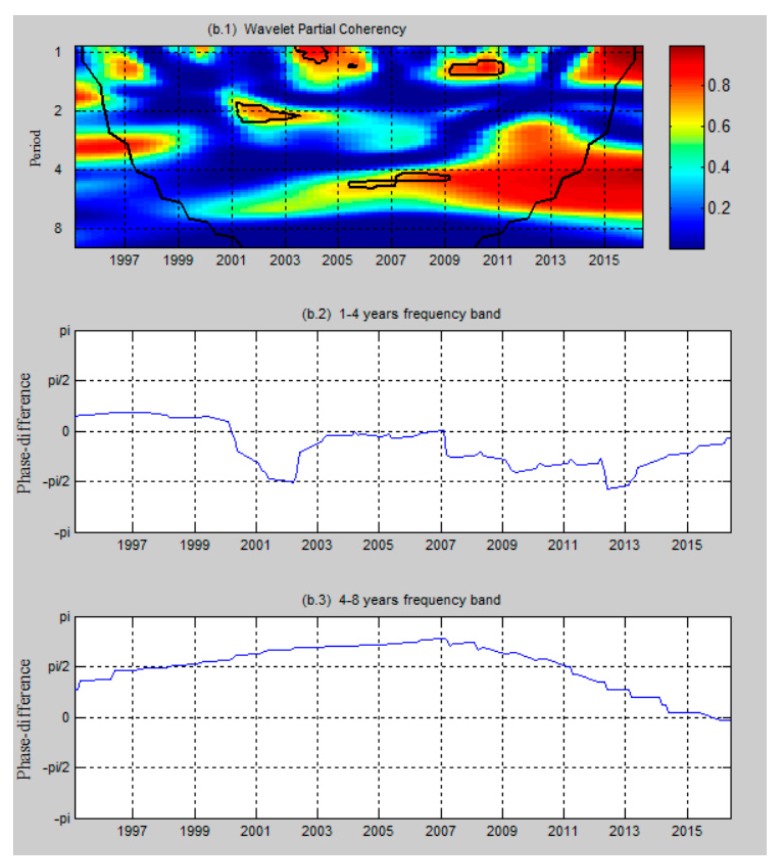
The partial wavelet coherency (**b.1**) and partial phase difference (**b.2**,**b.3**) between per capita CO emissions and healthcare expenditure per capita, with real GDP per capita as a control variable. The *y*-axis refers to the frequencies; the *x*-axis refers to timespan 1995–2016.

Figure 4 reports the wavelet coherency and the phase difference between per capita CO emissions and healthcare expenditure per capita in Taiwan, while Figure 5 shows the results when we add real GDP per capita as a control variable. Wavelet analysis can be performed even if the underlying series are non-stationary or are locally stationary [35]. No matter if the time series of the aforementioned variables are either non-stationary or stationary, wavelet analysis is adapted to reveal the lead-lag relationship. Thus, the unit root test is omitted in this part. In the figures, co-movement and causality are absent in the condition that the correlated regions drop to the inner of cones of influence (COI) and apart from the ninety percent confidence interval. A 95 percent confidence interval estimated from Monte Carlo simulations using a phase-randomized surrogate series exists when thick black lines appear in the wavelet coherency. As shown in the figures, the co-movement and causality between per capita CO emissions and healthcare expenditure per capita show substantial time- and frequency-variations in Taiwan, while time- and frequency-variations are also significant when we add economic growth as a control variable. Also, there is a distinct rise in the co-movement after the policy in environmental pollution is executed, which suggests that the policy in environmental pollution has led to a significant impact on the relationship between CO emissions and healthcare in Taiwan. Moreover, other events, such as the issue of healthcare certification authority IC cards and the period of the expansion stage after the recession, have impacted the co-movement.

Figure 4 reports the relationship between per capita CO emissions and healthcare expenditure per capita in the period 1995–2016. As shown, per capita CO emissions and healthcare expenditure per capita significantly co-move across frequency bands of 0.9–1.1, 0.9–1.25 and 1.75–2.2 years. In the short term, we focus on the 0.9–2.2 year frequency band. The results indicate positive co-movement scattered for the following periods: 1998–1999, 2001–2003 and 2006–2013. The coherency for the aforementioned periods is around 0.85. The phase difference of (−π/2,0) indicates that per capita CO emissions and healthcare expenditure per capita positively co-move over the period of 1998 to 1999 and 2001 to 2003 on one hand, and on the other, there is causality from healthcare expenditure per capita to per capita CO emissions before 2005. Per capita CO emissions and healthcare expenditure per capita move together in the short term when the phase difference is around zero. In other words, no sufficient evidence shows lead–lag relationships between two variables. Additionally, in the short term, from 1999 to 2007 and from 2011 to 2013, co-movement exists in the 6-to-7-year frequency band. The coherency for the aforementioned periods is around 0.95 in the interval (π/2,π). This observation suggests that healthcare expenditure per capita and per capita CO emissions strongly and negatively co-move, and there is unidirectional causality from healthcare expenditure per capita and per capita CO emissions in the long term.

The intuition behind such short-term positive causality is that higher government health expenditure positively affects economic performance, for the reason of positive externalities and conflicts between individual and public benefits [48]; also, EKC theory shows the positive impact of economic growth on the environment. Thus, we conclude that when the economy is in a developing stage or an expansion stage in terms of the economic cycle, health expenditure may positively impact environmental pollution.

After the government introduced the scheme of National Health Insurance in 1995, users, no matter the rich or poor, were able to enjoy the resources in the scheme in which users can undergo health examinations and cures with partial subsidies from the scheme, such that National Health Insurance benefitted everyone in the society. The NHI’s benefits are comprehensive; for example, the insurance includes the fees of inpatient care, daycare for the mentally ill, limited home health care, certain preventive medicines and even expensive treatment for diseases such as HIV/AIDS [49]. The government often subsidises those who are not rich in society; for example, in Taiwan, farmers are endorsed 70% and low-income families are endorsed 100% for the payment [22]. As a result, those users who cannot relatively afford huge medical fees benefit from the scheme. Thus, the people in the middle or bottom of the economic ladder, who now possessed better physical and mental situations, contributed to the economy, such that the positive externalities in the awareness of quality of life were shown after the introduction of the scheme. The above description shows that healthcare expenditure per capita lead to per capita CO emissions in the long run.

Government spending on healthcare has increased year by year as a result of overuse of medical resources by individuals; for example, users may often perform medical examinations instead of paying attention to their health situations in advance, and the scheme may be taken as the Tragedy of the Commons [49]. In 2003, an IC card policy was introduced and this policy implies that the prevalence of “hospital shopping” has become more serious because patients can easily access nearly all healthcare institutions with their NHI-IC cards [50]. The degree of spending on those who need to be subsided has decreased, and thus, the degree of externalities does not significantly perform. Thus, after NHI-IC cards were introduced in 2003, it did not show significant lead–lag relationships between healthcare expenditure per capita and per capita CO emissions in the short run.

Figure 5 reports the relationship between per capita CO emissions and healthcare expenditure per capita, taking economic growth as the control variable in the period 1995–2016. The causality between per capita CO emissions and healthcare expenditure per capita indicates a distinctive pattern when economic growth is taken as the control variable. From 2001 to 2002, the phase difference around −π/2 indicates that there is no sufficient evidence to support the existence of lead–lag relationships between per capita CO emissions and healthcare expenditure per capita, and the aforementioned variables move together during this period. In the periods 2002–2003 and 2009–2011, we see positive co-movement at the 1.8–2.2- and 1.2–1.4-year frequency bands. The coherency for aforementioned periods is around 0.7 and 0.8, respectively; it means that a short-run causality running from healthcare expenditure per capita to per capita CO emissions, showing that healthcare expenditure per capita leads per capita CO emissions. When the frequency band is around 4 to 4.4 years, the results statistically support co-movement between the aforementioned variables for the timespan 2006–2009. The phase difference is located within the interval (π/2,π), with an increased coherency of 0.7, indicating that healthcare expenditure per capita and per capita CO emissions are negatively related, and healthcare expenditure per capita has a relatively stable causal effect on per capita CO emissions during this period.

After we add economic growth as a control variable, the stage of expansion in the economic cycle, i.e., the period of 2002 to 2003 and 2009 to 2011, in Taiwan shows that the positive impact of healthcare expenditure per capita on per capita CO emissions. Thus, we confirm that the contribution of externalities to economic growth depends on the stage of the economic cycle. In the period of 2001 to 2002, Taiwan was in recession or early recovery, such that healthcare expenditure per capita and per capita CO emissions do not show significant lead–lag relationships. However, in the period of 2002 to 2003 and 2009 to 2011 in the short run, Taiwan was in an expansion or developing stage, such that healthcare expenditure per capita positively impact on per capita CO emissions based on the applications of positive externalities [48] and EKT theory.

The intuition behind such long-term negative causality is the following two perspectives and is related to the Armey curve. (Chen and Lee [51] indicated that threshold effects exist between government size and economic growth in Taiwan; thus, a non-linear Armey curve, as proposed by Armey [52] and Vedder and Gallaway [53], exists in Taiwan.). First, Chaabouni et al. [23] showed that the effect of health expenditure on the environment is negative and they take health expenditure as government size (government size may decline prosperity; consequently, it may decrease and increase pollution in certain conditions, based on the theory of EKC [48,54,55,56]) to explain the outcome of the environment.

Although the government may increase public expenditure, the performance of the economy may not increase because of several reasons, including crowding-out effects on the private sector, inefficiencies in government, and interventions on markets [54,57,58]. Bergh and Karlsson [55] and Afonso and Jalles [59] indicate that government size is negatively related to the economy. Thus, higher government health expenditure is more likely to impede economic growth for the reason of crowding-out effects on the private sector, and thus, decrease the severity of environmental pollution in the long-term.

Second, higher government health expenditure is more likely to include reallocation. It may lead to income equality. Consequently, well-being is pursued in terms of a healthy environment, and thus, higher demand for environmental quality is needed. As the environment is seen as a luxury public good, the environment comes under consideration once the demand for other public goods has been satisfied; that is, when society is at large levels of government size [60].

The launch of NHI in 1995 and the implementation of the total amount control in air quality policy in 1999 show that the government has put in the effort to decrease inequality for residents and establishing a better environment for life in the long-term. Also, the adequacy and equity for those who need access to health care is one of the goals in the NHI scheme. As a result, we find that people with sufficient wealth in society and the economy are more eager to pursue higher life quality, and the phenomenon was more significant in the period of 2005 to 2007 and 2011 to 2013. The result is consistent with Frederik and Lundström [60] and we support the environment as a luxury public good to be considered when the demand for other public goods has been satisfied.

After we add economic growth as a control variable in the long term, the period 2006–2009 shows the negative impact of healthcare expenditure on the environment. Compared to the pattern without the economy as the control variable, pattern with the economy as the control variable is significant in the period around 2008. It shows that it is an important structural breakpoint in 2008 in explaining healthcare–environment causality; also, economics is an important factor between the relationships. Thus, the results show that the consciousness of environmental protection issues are more observed in prosperity. In sum, the empirical results indicate that the causality runs positively from healthcare expenditure to environment in the expansion or developing stage in the short-term, while negative causality exists in the long-term.

## 5. Conclusions

This study contributes to the literature in the dynamic relationship among environmental degradation, healthcare expenditure and economic growth. We investigate the lead–lag relationship between environmental degradation, healthcare expenditure, taking economic growth as the control variable over the period of 1995 to 2016 in Taiwan by wavelet analysis. The results show that wavelet analysis is useful in evaluating the effects of different socio-economic scenarios in the context of different positions for healthcare and environment policies in Taiwan.

In the short-term, the causality runs positively from healthcare expenditure to the environment in an expansion or developing stage and it is consistent with the results of a prior study [24]. We support that economic growth as a bridge to link healthcare expenditure to the environment depends on the stage of the economic cycle. From the results, long-term negative causality running from healthcare expenditure to the environment exists and it is consistent with the results of Chaabouni [23]. Thus, we confirm that the government has put effort in decreasing inequality for residents and establishing a better environment for life in the long-term. In addition, the result is consistent with Frederik and Lundström [60] and we support that the environment is a luxury public good to be considered when the demand for other public goods has been satisfied.

Taiwan is taken as an emerging country and the result of the dynamic relationship between environmental degradation and healthcare expenditure in this study is not consistent with the existing argument of studies that investigate the dynamic relationship for developed countries. In Apergis et al. [25], they showed the causal effect runs from environmental degradation to healthcare expenditure in U.S. states, while the causality running from healthcare expenditure to environmental degradation exists in Taiwan in this study. The result in this study does not support the direction of causality of the prior study and the disparity may contribute to the stability of economics and policies on healthcare and the environment, depending on the development of a country. However, the socio- and economic implications behind the results are similar. For example, although the causality positively runs from healthcare expenditure to environmental degradation in Taiwan, the Taiwanese government should not expect that decreasing the healthcare expenditure results in an improvement of the environment. According to prior studies, the health of the population is fundamental in economics [9,61] by the contribution of human capital and corporate capital [62]. Also, the adequacy and equity for those who need access to health care is one of the goals in the scheme. The economics and society for emerging countries such as Taiwan are not as sophisticated as in developed countries such as the U.S. Therefore, government intervention is considered to bring better economic and social benefits to populations in Taiwan.

In Taiwan, health expenditure positively affects economic performance and it may contribute to positive externalities of government issues related to healthcare and environment policies to decrease the conflicts between private and social interests. In addition, when the economy is in a developing or expansion stage in Taiwan, health expenditure may positively impact environmental pollution. After introducing NHI, the people in the middle or bottom of the economic ladder, who now possess better physical and mental situations, contributed to the economy, such that the positive externalities in the awareness of quality of life were shown. It showed significant lead–lag relationships between healthcare and the environment until NHI-IC cards were introduced in 2003. Therefore, the prevalence of “hospital shopping” is more serious and NHI may be taken as the Tragedy of the Commons. The government in Taiwan is devoted to decrease inequality for residents and establishing a better environment for life based on the launch of NHI and the implementation of environmental policies. Based on empirical analysis in this study, policymakers may create beneficial healthcare and environmental policies that are addressed to decision-makers at the macroeconomic and microeconomic levels. The causality positively (negatively) runs from healthcare expenditure to the environment in the short-term (long-term). This empirical result indicates the launch of the NHI not only shows that the government has put the effort to decrease inequality for residents but also to help the people who possess better physical and mental situations in the middle or bottom of the economic ladder contribute to the economy, such that the positive externalities in the awareness of quality of life are shown in the long-term. The direction of causality between healthcare expenditure and the environment is different in Taiwan and the U.S. states [25]. Taiwan is one of the few emerging countries that implement an NHI subsided by government spending. The result is helpful for the government to understand policy implications in terms of the funding and distribution of healthcare resources in the NHI scheme. In sum, this study is helpful for policymakers for healthcare and environmental policies in Taiwan; this study also serves as a reference point for policymakers in other similarly developed economies such as Taiwan, in short or long horizons. Finally, since the funding of the NHI scheme is mainly subsidised by the government, it is suggested that future work takes into account government spending to assess economic growth and the environment by using time-frequency domain techniques to comprehensively understand the impacts of policy implementation.
